# Vacant Appointments : Should Canvassing Be Encouraged or Forbidden?

**Published:** 1914-12-05

**Authors:** 


### THE EDITOR'S LETTER-BOX.

### Vacant Appointments.

### Should Canvassing be Encouraged or Forbidden?

*To the Editor of* The Hospital.

Sir,—*Apropos* of the comments in The Hospital of the 26th inst., it cannot be meant seriously that any organisation, be it the British Hospitals Association or the Incorporated Association of Hospital Officers, really advocates that advertisements inviting candidates for any appointment shall contain a clause that canvassing is prohibited. Presumably the object of an advertisement is to obtain the best man to fill the vacant office. Is it possible to attain this object if candidates are not seen beforehand? And is there any possibility of preventing the deception that is so justly censured?

No elector who hopes to do his duty can properly exercise the powers he is vested with unless he has an opportunity of seeing the candidates before the dread moment when they appear at the board-room table. No man appears at his best under such circumstances; all are at a disadvantage. The thoughtful, conscientious candidate is at his worst, while self-assurance and effrontery, so often coupled with incapacity, have a good chance to carry the day. There is not one elector in a hundred who has the power or the perspicacity to select the best man out of a series of candidates appearing for a few minutes amid strange surroundings in the gloom of the average committee room. It is not fair either to the candidate or to the elector. The latter should be afforded the opportunity to see and hear candidates. Five minutes' straight talk with a man will give a better insight into his character and capacity than ten interviews with a committee. Electors should be encouraged to inquire about the men, to get the views of those who know them and who have worked with them, and should not be contented with the flamboyant and deceptive testimonials that the writer of the article appears to think sufficient to guide the elector in throwing in the weight of his vote and interest to place in office a man who may be utterly unfit to carry out the responsible duties of a public official. If electors would interview candidates beforehand, we should see less of the unsuitable and sometimes incapable men trying to administer public institutions. Hospitals perhaps more than other charities are dependent on the ability of permanent officials. The results of incapacity must be obvious to anyone who knows the working of the voluntary system, and once the man is appointed it is difficult to dislodge him.

There can be little doubt that the insertion of a paragraph in an advertisement forbidding canvassing is accountable for some, at any rate, of the ineptitude that hangs about and retards the growth of some of the medical charities. The elector who thinks it too much trouble to see candidates has no right to take the responsibility of a committeeman upon him. The evil of this apathy is seen every day in the minor appointments, and those who have worked for any length of time on committees for the appointment of senior resident medical officers are aware how often the best candidate is left among the rejected simply because the members of the Medical Council won't be disturbed to see the candidates before the day of election.—Yours faithfully, A Hospital Secretary.

November 27, 1914.

[Our correspondent's interesting letter misses the point of our paragraph, which was to show that where committees have issued advertisements prohibiting canvassing, as men of honour and men of sense they must see that this prohibition is adhered to. His remark that "the elector who thinks it too much trouble to see candidates has no right to take the responsibility of a committeeman upon him," is one with which we cordially agree. But whether this is an argument for canvassing or against electing deadheads to committees is open to doubt. In any case, if the prohibition is inserted it must be acted upon, in fairness and honour to all candidates.—Ed. The Hospital.]

### Discipline in Sanatoria: The Grumbles of Working-Class Patients.

*To the Editor of* The Hospital.

